# More than just needles: An evidence-informed approach to enhancing harm reduction supply distribution in British Columbia

**DOI:** 10.1186/1477-7517-5-37

**Published:** 2008-12-24

**Authors:** Jane A Buxton, Emma C Preston, Sunny Mak, Stephanie Harvard, Jenny Barley, BC Harm Reduction Strategies and Services Committee

**Affiliations:** 1 Epidemiology Services, British Columbia Centre for Disease Control, 655 West 12th Avenue, Vancouver, Canada; 2 School of Population and Public Health, University of British Columbia, 5804 Fairview Avenue, Vancouver, Canada

## Abstract

**Background:**

The BC Harm Reduction Strategies and Services (HRSS) policy states that each health authority (HA) and their community partners will provide a full range of harm reduction (HR) services to their jurisdictions and these HR products should be available to all who need them regardless of where they live and choice of drug. Preliminary analysis revealed wide variations between and within HAs.

**Methods:**

The objective of this study is to analyze distribution of HR products by site using Geographic Information Systems (GIS) and to investigate the range, adequacy and methods of HR product distribution using qualitative interviews. The BC Centre for Disease Control pharmacy database tracks HR supplies distributed to health units and community agencies. Additionally, eleven face-to-face interviews were conducted in eight mainland BC communities using an open-ended questionnaire.

**Results:**

There is evidence in BC that HR supplies are not equally available throughout the province. There are variations within jurisdictions in how HR supplies are distributed, adequacy of current HR products, collection of used needles, alternative uses of supplies and community attitudes towards HR. GIS illustrates where HR supplies are ordered but with secondary distribution, true reach and availability of supplies cannot be determined.

**Conclusion:**

Currently, a consultant is employed to develop a 'best practice' document; relevant health files, standard training and protocols within HAs are also being developed. There is a need to enhance the profile and availability of culturally appropriate HR services for Aboriginal populations. Distribution of crackpipe mouthpieces is being investigated.

## Background

The British Columbia (BC) Harm Reduction Strategies and Services (HRSS) committee has representation from each of the 5 regional health authorities, the BC Ministry of Health and the BC Centre for Disease Control (BCCDC). The BC HRSS policy states that each health authority and their community partners will provide a full range of harm reduction (HR) services to their jurisdictions and that the HR products should be available to all who need them regardless of where they live and choice of drug [1]. The HR products distributed include condoms and lubricants, needles and syringes, alcohol swabs and sterile water and are funded by the BC Ministry of Health and subsidized by the Provincial Health Services Authority.

The HR product distribution is coordinated by BCCDC; the BCCDC pharmacy database tracks HR products ordered by health units and community agencies (approved by the health authorities) that distribute the supplies. Over 20 products are currently available for distribution to the more than 150 ordering sites in BC. Preliminary analysis of the data revealed wide variations between and within health authorities. As a result of these discrepancies we identified a need to evaluate current product supply distribution, identify gaps, cost-saving measures and potential future demands.

The objective of this study is to:

1) Analyze distribution of HR products by site using geographic information systems

2) Investigate the range, adequacy and methods of HR product distribution using qualitative interviews.

Much of the current information and knowledge surrounding HR in BC is derived from Vancouver; therefore we sought to include the perspectives of distribution sites outside Vancouver.

## Methods

Product distribution by site was obtained from the BCCDC pharmacy database. We used a period of 19 months (May 2006-November 2007) to ensure inclusion of sites that placed infrequent orders i.e. less than annually. All needles with syringes attached (0.5 and 1 cc) and individual needles (but not individual syringes) were collated to produce the total volume of needles distributed and were analyzed using geographic information systems.

Interview sites were selected purposively from BCCDC pharmacy database to ensure a range of geographic factors and volume of supplies distributed. An invitation letter was sent to the contact at each selected site. A research assistant contacted potential participants to arrange an approximately one-hour in-person interview.

The semi-structured interviews consisted of open-ended questions developed by the research team. The questions were modified to explore emerging concepts as data collection progressed [2]. Interviews were audio-taped and the research assistants made field notes of their observations.

Questionnaire domains included:

1) How HR supplies are distributed

2) Perspectives on the adequacy of current harm reduction products

3) Collection of used needles

4) Alternative uses of supplies

5) Perceived community buy-in

The interviews were transcribed verbatim and analysed using standard qualitative methods. Members of the research team reviewed the transcripts and independently identified themes within the pre-determined domains and from open-ended comments. Transcripts and field notes were reviewed in an iterative manner to ensure all emergent themes were captured. Representative quotes were selected from the transcripts to illustrate the main themes identified.

To inform the findings, the mapping and qualitative analysis were presented to HRSS committee members for further input; notes of the discussions were taken. Ethical approval was received from the University of British Columbia Behavioural Research Ethics Board.

## Results

### Supply distribution

Supply orders were tabulated into reports to illustrate date and quantity of each category of products ordered by each individual site, collated into 5 regional health authorities and the 16 health service delivery areas in BC. Input was received from HRSS committee regarding the report format and utility. Committee members agreed to use the information to provide feedback to their health authorities and distribution sites with regard to appropriate ordering frequency and product quantity to improve fiscal responsibility. Some sites supplied only condoms; others provided a full range of products. Single use water vials ordered varied from 0% – 70% of quantity of needles supplied.

**Figure 1:**
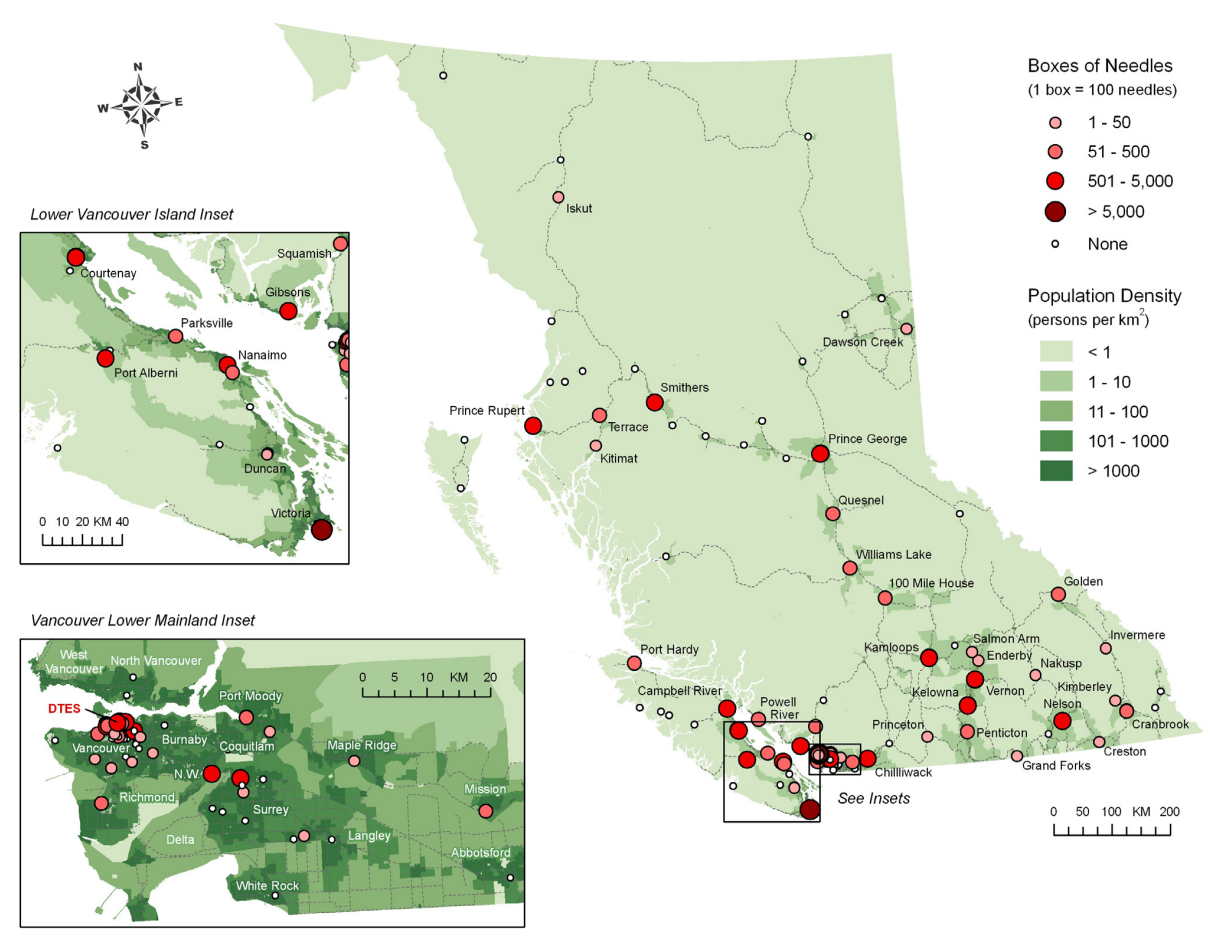
**Distribution of needles and syringes in British Columbia May 2006–November 2007**.

Figure 1 shows the results of geographic information system mapping of the distribution of needles and syringes in the province of British Columbia between May 2006 and November 2007. Each dot represents a site where harm reduction supplies are ordered and distributed through public health nursing and other community health organizations. The smaller white dots represent communities where harm reduction supplies are distributed but not needles and syringes (i.e. condoms only).

### Qualitative interviews

Eleven face-to-face interviews were conducted in eight mainland BC communities. All selected interview sites agreed to participate. Interviews occurred with providers at health units, community health centers, Aboriginal Youth and Friendship Centers, and HIV/AIDS agencies and organizations; three of the interview sites did not distribute needles.

#### 1) How HR supplies are distributed

The themes that emerged included: a) variations in how the supplies were made available to the clients and the degree of client engagement; b) one-for-one needle 'exchange' versus a needs basis distribution; c) data collection and d) trends in demand. Availability of supplies was item and site dependent. Some health units reported distributing sex products only, as injection supplies were available from a nearby agency. Some sites have condoms in a basket at the reception desk and in washrooms so clients can help themselves; other sites required clients to ask for all supplies, which were provided by the receptionist or the nurse on call. One site requested the client to call ahead to place their order in advance. A few sites provided harm reduction items in brown bags; clients selected bag A or B from a list or picture, which showed number of items in each, depending on their needs. A number of the health units had designated rooms in which supplies were stored and where the client met privately with the provider to obtain supplies and return used needle.

The degree of client engagement was highly variable. Some providers routinely engaged clients and reported regular referrals to 'detox' or clinics for sexually transmitted infections and blood borne pathogen testing. No standard protocols or training of the HR supplies providers were reported to be available in the rural sites.

All but one respondent reported giving supplies to individuals or agencies for distribution at that site i.e. "*secondary distribution*." Although individuals from First Nations communities obtain their supplies from the provider sites, no supplies were reported to be obtained for secondary distribution on reserve by nurses or other representatives. One site reported a female who came in for supplies to take to the working girls and at other sites clients took supplies in large quantities to share.

We have a regular exchange user who is male. And he's been coming for years and he exchanges for his group as well

All sites encouraged needle *exchange*; but only one site reported trying to ensure one-for-one exchange. However, even this site supplied a single clean needle and sometimes 4 or 5, even if no needles were returned. This was perceived to prevent people from 'tossing' needles and encouraged people to collect discarded needles found on the ground to exchange for clean ones.

Data collection also varied considerably from site to site. No systematic data collection of supplies obtained for "secondary distribution" was reported. One site registered individual clients by birthday; this site also tracked demographic information, drug of choice, HIV testing etc. Other sites collected no client information and had no tracking system.

We don't collect any demographic information from clients in any way. It is supposed to be anonymous

The demand for supplies fluctuated. For example, the demand for needles was reported to be highest around the time welfare checks were issued. A large lower mainland site reported a considerable decrease in needle distribution over time; it was estimated the number of needles distributed per month had almost halved to 15,000–18,000 a few years ago when smoking crack cocaine became the drug of choice. Although some rural sites reported a decline in needle distribution others noted a steady increase in demand as '*word got around'*.

#### 2) Perspectives on the adequacy of harm reduction products

This section discusses input regarding current supplies, by item category, and then will explore what is perceived to be missing from the list. Male condoms were available at each site; lubricated condoms were generally preferred to non-lubricated and one distributor reported providing in a ratio of about 5:1. Clients usually did not specify a preference of condom type although younger clients preferred flavoured condoms.

Female condoms were not widely used, some sites required women to ask specifically for them, as they believed this ensured provision of adequate education regarding use. The two sites with the greatest distribution reported actively engaging the women and teaching about female condom use. One site sent the female clients to a clinic next door as

...... *this is a good way to get the girls 'checked over*' [tested for sexually transmitted infections]

Most clients use 0.5 or 1 cc syringes with needles attached. Larger syringes and needles were reportedly used for injecting steroids. There was general consensus that clients were not using sterile water for every injection, though some sites thought the demand for water was increasing.

The demand of water is not comparable, in terms of, people will take more needles than they will take water, in fact we ask them specifically every time they... ask for needles, do you want water?

One site reported distributing no sterile water

We don't ever get asked for water... It's just the needles

Requests for additional supplies include those used for injecting drugs e.g. cookers, filters, tourniquets and sharps containers; miscellaneous e.g. paper bags in which to hand out supplies and drinking water for clients, and finally those related to crack use e.g. crack pipes, mouthpieces and screens. Crack was perceived as the most commonly used drug in many of the areas, and that an increasing number of clients were asking for crack smoking paraphernalia. Some sites reported purchasing their own additional items for injection or crack use.

#### 3) Collection of used needles

All sites reported encouraging clients to return used needles.

Users bring in used needles and...we have a large sharps container that they put them into

Some sites provided clients with individual sharp containers, which varied between official yellow biohazard containers to empty rigid shampoo bottles. Sites distributing sharps containers requested that they be returned to the provider site when full. Others stated that clients reported concern about collecting and keeping needles in the home when there were children in the household.

#### 4) Alternate uses of HR supplies

Condoms had a number of different alternate uses. Non-lubricated condoms were reported to be used as tourniquets for injection drug use, and also by crack smokers who hold exhaled smoke in the condom to share or inhale it '*for a second take*'. One site removed the condom basket from the front desk and washrooms in the summer as teenagers were using them as water balloons, leaving broken condoms on the sidewalk outside the office.

Providers in Vancouver revealed that the plungers of syringes were being used as a pusher for crack pipes to recover the crack resin dried on the inside of the pipe as it cools. When this was explored further with Vancouver front line staff it was estimated about 1 in 5 syringes were being used for this purpose, and the needle and barrel of the syringe discarded.

#### 5) Community buy in/readiness

Participants reported few community development initiatives regarding HR or pick-up of discarded needles. There was a perception that HR philosophy was new to many health care workers and the general public.

*The community with professionals and the public the flavour is currently stop the drug use. If we stop the drug use we could clean up the mess kind of thing... we all know... that doesn't work*.

However some interviewees felt their community was ripe to hear the messages because '*there's been a few drug related tragedies [recently]'*.

## Discussion

Availability of clean needles (via needle exchange programs) has been shown to decrease the rates of transmission of HIV and hepatitis C (HCV) [3]. A recent study found that full participation in HR programs, including methadone, could decrease the risk for HIV and hepatitis C [4]. Therefore it is important, as stated by HRSS policy, that HR supplies are available to all who need them. However, there is evidence in BC that supplies are not equally available throughout the province. Spittal et al found Aboriginal youth in Northern BC had more difficulty accessing clean syringes than Vancouver youth [5]. No official harm reduction distribution on First Nations reserves was reported. Several barriers to comprehensive harm reduction services for First Nations persons have been identified by Wardman et al. These include cultural differences, stigma, limited service infrastructure and financial resources, and community size [6] While the abstinence model for the treatment of addictive disorders is considered the norm in many First Nations communities, it is acknowledged that it is possible to enhance the profile and availability of culturally appropriate HR services in this context. This may include incorporating traditional Aboriginal practices, providing additional services such as education and counseling in conjunction with HR programs, and integrating into existing reserve public health programs [6,7]

Geographic information systems illustrated sites and the volume of HR supply distribution in BC, and by inference where availability may be lacking. However without secondary distribution information, the true reach and availability of supplies cannot be determined. Product distribution by population can be calculated for each health authority, but the variations within each jurisdiction are vast. It is interesting to note that Fraser Health with the largest health authority population in BC has only eight communities where supplies are delivered. Harvard et al found regional variations of BC harm reduction product distribution. However using reported HCV cases, as a proxy for injection drug use, variation in product distribution could not be attributed to variations of estimated prevalence of injection drug use [8].

Qualitative research seeks to explore process, opinions, attitudes and actions. It is the best method to answer questions about a topic, which may be sensitive and/or about which little is known. Sampling in qualitative studies is purposeful; so we explored the perspectives of HR distributors in sites outside Vancouver including rural areas. Qualitative interviews do not aim to be representative or generalizable; however we found recurrent common themes from different sites.

To improve the understanding of HR for health care providers and the public a generic 'Understanding Harm Reduction' health file [9] has been recently published. Despite the provincial policy of HR distribution on a needs basis, one site interviewed maintains one-for-one exchange. A health file discussing 'needle distribution vs. exchange and community engagement' is therefore in development.

Training of volunteers and staff to give HR advice and referrals for services and testing can increase client engagement. Sites where women received instruction on the use of female condoms distributed more of these items. Best practice guidelines suggest that distribution of needles and syringes should be comparable to the rates of sterile water, as both products should be used for every injection. However there is a wide variation in the request and offering of water for injection; some sites encouraged the use of water vials for each injection whereas others distributed no water because they were not asked for it.

Although flavored condoms were not in great demand it was felt important to continue, as these were popular with the younger population who should be encouraged to use safer sex products. Many sites requested sharps containers. However advice re safe collection of needles using rigid plastic containers such as shampoo bottles could improve the safety in the household and transportation and enhance needle return to the sites.

The use of syringe plungers to push the resin through the hot crack pipe, may lead to melting the plastic plunger and discarding of the needle and syringe barrel. The distribution of wooden push sticks through the HR supplies is currently being investigated. Clients at many sites requested crack pipes and mouthpieces. Two infectious disease outbreaks have been reported in BC associated with crack use. In 2006 an outbreak of Streptococcus pneumonia in the DTES of Vancouver was identified, [10] and a Tuberculosis outbreak in a crack using population was reported elsewhere in BC [11]. A recent study detected hepatitis C virus on a crack pipe from an infected host, and therefore supports the possibility of transmission through sharing crack paraphernalia [12]. Crack users may have open mouth sores due to burns and cuts from hot and broken pipes therefore sharing crack pipes can transmit respiratory infections and blood-borne pathogens, including HCV and HIV [13]. Crack pipe mouthpieces are now available through the provincial BC HR supplies and each HA is undergoing consultation to determine if, and how, to provide these.

One HA has developed a training module; all urban site providers must participate in the training before they can distribute supplies, and is willing to share with other regions. Standard training and protocols within health authorities can lead to improved client engagement and awareness of the client needs. It may also encourage peers to be involved in distribution and needle collection. Community engagement is uncommon in rural areas, regions that have developed the process can share their experiences and lessons learned to enhance public understanding of harm reduction.

The mapping of needle distribution sites provides a highly visual way to show the limitations of primary distribution sites and enables health authorities to assess the reach of supplies in their regions. The qualitative research highlighted the lack of standardization between and within each health authority in BC. Therefore a consultant has been employed to develop a 'best practice' document to assist regions in employing standardized evidence-based process and protocols to improve access of supplies and client and community engagement. Development of a secondary distribution data collection tool and sharing of training modules will be explored. Additionally, as this work is continued it is critical that the risk environment is taken into account in order to address issues at the community level and create 'enabling environments' for harm reduction [14].

## Conclusion

This study has contributed to the evidence that HR supplies are not equally available throughout the province of British Columbia. The use of GIS in this study illustrates where availability of HR supplies may be lacking. However; with secondary distribution, true reach and availability of supplies cannot be determined. Variations within jurisdictions must also be taken into consideration. Development of standard training and protocols within HAs will play a important role in ensuring optimal utilization of HR supplies through BC and will lead to increased client awareness and engagement. Additionally, further research is needed to gain a better understanding of HR supply distribution, to enhance the profile and availability of culturally appropriate HR services for Aboriginal populations, and to create enabling environments for harm reduction across the province.

## Competing interests

The authors declare that they have no competing interests.

## Authors' contributions

JBu is the primary investigator for this study and was involved in the interview analysis and manuscript writing. Emma Preston contributed to the qualitative interviews and manuscript writing. SM was responsible for the GIS analysis. SH performed qualitative interviews and aided in manuscript writing. JBa reviewed qualitative interviews and assisted in manuscript writing. All have read and approved the final manuscript.
